# Cyclooxygenase-2 up-regulates hepatic somatostatin receptor 2 expression

**DOI:** 10.1038/s41598-018-29349-y

**Published:** 2018-07-23

**Authors:** Yao-Yao Lu, Jin-Hang Gao, Chong Zhao, Shi-Lei Wen, Cheng-Wei Tang, Yu-Fang Wang

**Affiliations:** 1 Division of Peptides Related with Human Diseases, State Key Laboratory of Biotherapy, West China Hospital, Sichuan University, Chengdu, China; 2 Department of Gastroenterology, The First Affiliated Hospital of Chengdu Medical College, Chengdu, China; 3 Department of Gastroenterology, West China Hospital, Sichuan University, Chengdu, China; 4 Division of Digestive Diseases, West China Hospital, Sichuan University, Chengdu, China; 5 Department of Human Anatomy, Academy of Preclinical and Forensic Medicine, West China Medicine College, Sichuan University, Chengdu, China

## Abstract

Somatostatin and its analogues, which function by binding to somatostatin receptors (SSTRs) 1–5, play a protective role in liver cirrhosis. Hepatic SSTR-2 expression is up-regulated in subjects with liver cirrhosis. However, little is known about the mechanisms underlying this process. In the present study, we observed the up-regulation of hepatic SSTR-2 expression in thioacetamide (TAA)-induced cirrhotic rats and further showed that cyclooxygenase-2 (COX-2) might play a role in this process via the protein kinase C (PKC)–cAMP response element binding protein (CREB) signaling pathway. *In vivo*, the up-regulated SSTR-2 in liver cirrhosis was inhibited by the addition of a selective COX-2 inhibitor, such as celecoxib. *In vitro*, the up-regulation of COX-2 by either transfection with COX-2 plasmids or treatment with TAA increased levels of SSTR-2 and phosphorylated CREB (p-CREB) in the human hepatocyte cell line L02. Furthermore, the increase in SSTR-2 expression was inhibited by the addition of celecoxib and a PKC inhibitor. Moreover, for comparable DNA methylation levels in the region upstream of the hepatic SSTR-2 gene in normal and cirrhotic livers, DNA methylation may not contribute to the up-regulation of SSTR-2 expression in cirrhotic livers. In conclusion, the up-regulation of hepatic SSTR-2 might be induced by COX-2 via the PKC-CREB signaling pathway but is probably not induced by DNA methylation.

## Introduction

Somatostatin (SST) is a polypeptide that is widely distributed throughout the human body. SST has been demonstrated to mediate a wide range of physiological functions since it was initially shown to inhibit the secretion of growth hormone. SST inhibits cell proliferation and growth, enhances apoptosis, and inhibits digestive track motility. Moreover, SST acts as a neurotransmitter and neurohormone in the nervous system^1,2^. Additionally, SST and its analogues protect against liver cirrhosis^3^. The SST analogue octreotide alleviates liver cirrhosis by directly inhibiting the synthesis of alpha-smooth muscle actin (α-SMA) and collagens in hepatic stellate cells (HSCs)^4–6^. Furthermore, SST and its analogues reduce splanchnic blood flow and decrease portal vein pressure by inducing the contraction of peripheral vessels, inhibiting the secretion of vasodilatory peptides and the contraction of HSCs^7–9^. SST functions by binding to its G-protein-coupled receptors, somatostatin receptors 1–5 (SSTR-1–SSTR-5). Of the five receptors, SSTR-2 exhibits the most diverse functions. According to previous studies, the hepatic expression of SSTRs, particularly SSTR-2, is up-regulated in subjects with liver cirrhosis^10^. However, the regulatory mechanisms involved in this process remain obscure.

DNA methylation at CpG islands is a common epigenetic modification in eukaryotes. CpG islands are defined as DNA fragments containing a large number of CpG dinucleotides^11^. Usually, DNA hypermethylation at CpG islands in the promoter area inhibits gene expression^12–14^. SSTR-2 levels in several tumor cell lines are negatively correlated with the DNA methylation status in its promoter region, and the DNA methylase inhibitor 5-aza-deoxycytidine up-regulates SSTR-2 expression^15–17^. However, researchers have not determined whether DNA methylation at the SSTR-2 promoter region contributes to the up-regulation of SSTR-2 expression in the cirrhotic liver.

SSTR-2 expression has been reported to be up-regulated under inflammatory conditions, such as IgA nephropathy, adipose tissue inflammation and ileum inflammation^18–20^. One study conducted in conjunctival and corneal epithelial cells found that inflammatory cytokines, such as interleukin (IL)-1α, IL-1β, lipopolysaccharide, and peptidoglycan, increased SSTR-2 expression^21^. Cyclooxygenase-2 (COX-2) catalyzes the synthesis of prostanoids from arachidonic acid and plays an important role in inflammation^22^. COX-2 is induced by various stimuli under inflammatory conditions. Specifically, as chronic inflammation ultimately leads to liver cirrhosis, the anti-inflammatory treatment of liver cirrhosis has received increasing attention^23–25^. In our previous study, significant up-regulation of COX-2 and SSTR-2 was observed in the livers of cirrhotic patients compared with the levels in non-cirrhotic patients^26^. Additionally, the COX-2 inhibitor celecoxib and the SST analogue octreotide synergistically ameliorate portal hypertension and liver fibrosis^3^. However, the relationship between the expression of COX-2 and SSTR-2 has not yet been investigated.

In the present study, we explored the effect of DNA methylation and COX-2 on SSTR-2 expression. COX-2 may contribute to the up-regulation of hepatic SSTR-2 expression in subjects with liver cirrhosis.

## Results

### Establishment of a liver cirrhosis model

**Figure 1:**
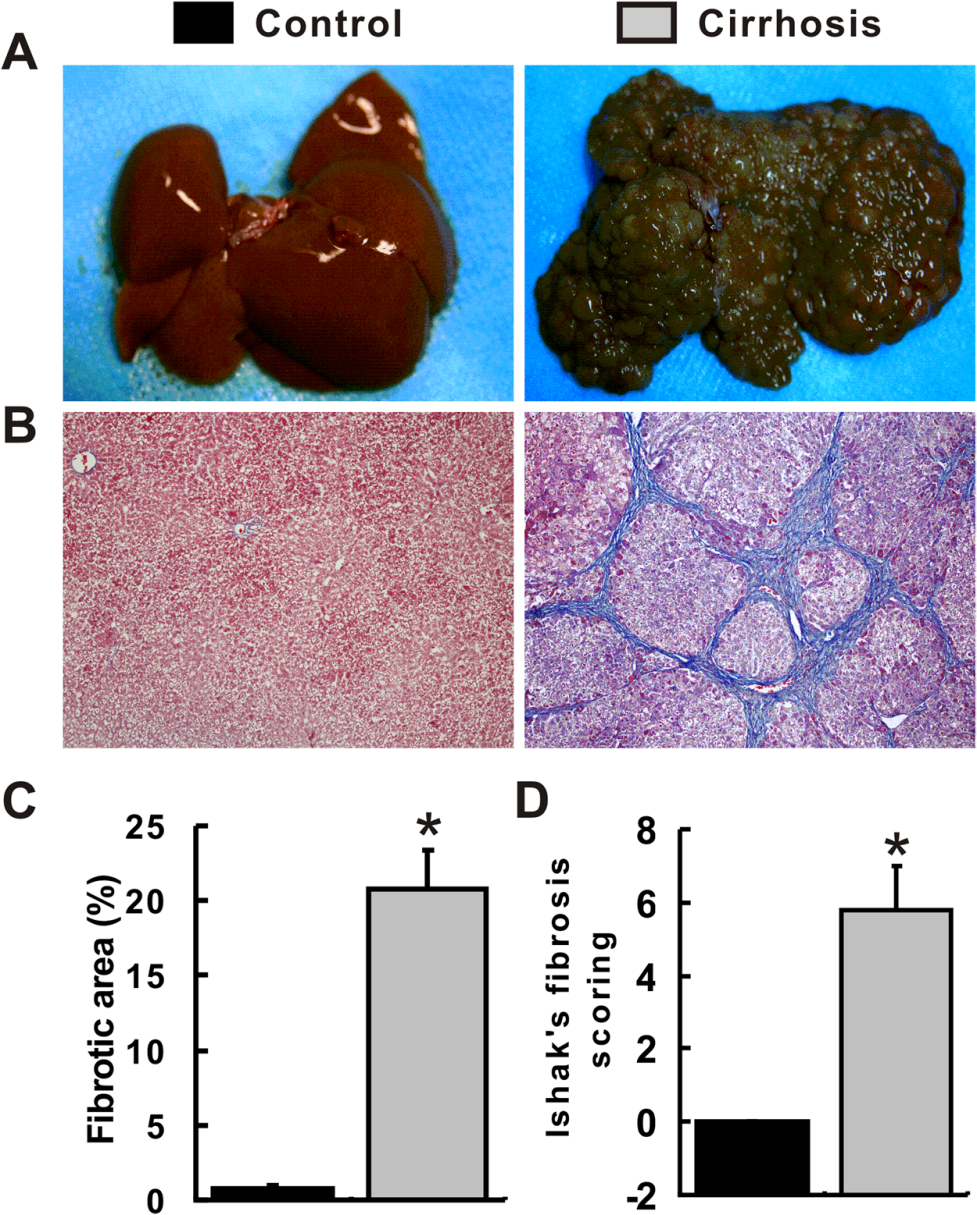
Establishment of a liver cirrhosis model. (**A**) Gross appearance of the liver. (**B**) MT staining of liver tissues. (**C**) Statistical analysis of fibrotic areas. (**D**) Statistical analysis of Ishak’s fibrosis scores. ^*^P < 0.05 compared with the control group. ^#^*P* < 0.05 compared with the cirrhosis group.

Compared with rats in the control group, rats in the cirrhosis group showed a lower body weight, duck, and lethargy. All rats receiving thioacetamide (TAA) developed liver cirrhosis, and none experienced toxicity-related death. Livers in the control group were generally tender and reddish, with a regular and smooth surface. However, livers in the cirrhosis group exhibited a hard texture, blunt edges, a brown color and a nodular surface (Fig. 1A). Consistently, typical liver cirrhosis was also observed in the cirrhosis group using Masson’s trichrome staining (Fig. 1B) and was characterized by the accumulation of extracellular matrix, destruction of the normal architecture and the formation of pseudolobules. Liver cirrhosis was further confirmed by the presence of fibrotic areas and Ishak’s fibrosis scoring. The fibrotic areas were significantly increased in the cirrhosis group compared with those in the control group (Fig. 1C). Ishak’s fibrosis score for all samples in the cirrhosis group was 5.8, compared with 0 in the control group (Fig. 1D).

### Up-regulation of SSTR-2 and COX-2 expression in the cirrhotic liver

**Figure 2:**
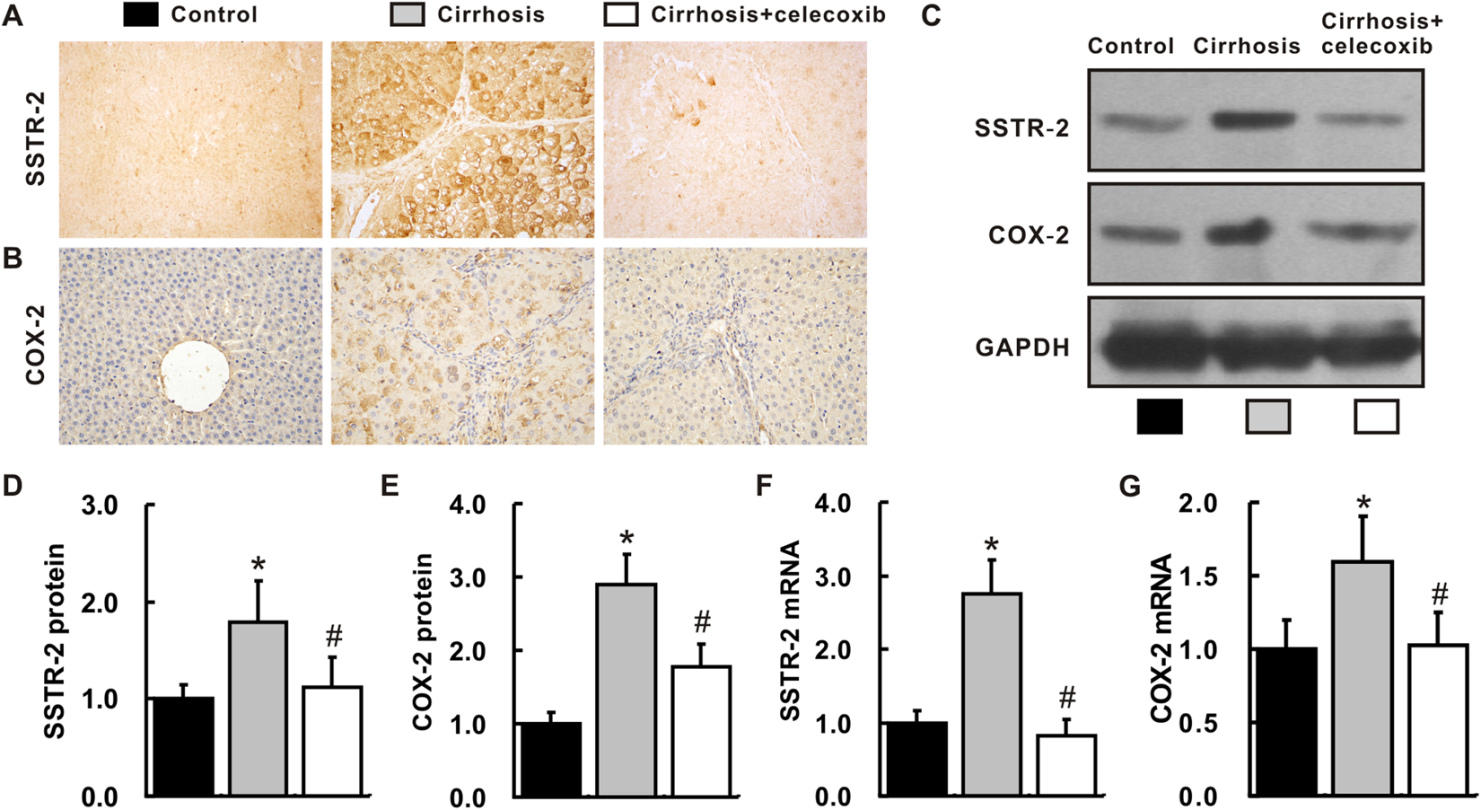
Up-regulation of SSTR-2 and COX-2 expression in cirrhotic livers. (**A**,**B**) Hepatic expression of the SSTR-2 (**A**) and COX-2 proteins (**B**) (IHC, magnification ×400). (**C**) Hepatic expression of the SSTR-2 and COX-2 proteins, as determined by Western blotting. The group of blots was cropped from different gels. (**D**,**E**) Quantitative statistical analysis of the Western blots examining the hepatic expression of SSTR-2 (**D**) and COX-2 (**E**). (**F**,**G**) Hepatic expression of the SSTR-2 (**F**) and COX-2 (**G**) mRNAs, as quantified by qRT-PCR. n = 6 samples per group. ^*^P < 0.05 compared with the control group, ^#^*P* < 0.05 compared with the cirrhosis group.

Levels of the SSTR-2 mRNA and protein were determined in the rat liver. As shown by immunohistochemical staining (IHC), Western blot and real-time PCR, SSTR-2 expression was up-regulated in the cirrhosis group compared with that in the control group (Fig. 2A,C,D,F). Similarly, levels of the COX-2 mRNA and protein were also increased in the livers from the cirrhosis group (Fig. 2B,C,E,G). Interestingly, the up-regulation of SSTR-2 and COX-2 expression in the cirrhotic liver was remarkably inhibited by the administration of celecoxib (*P* < 0.05). A significant difference was not observed between the cirrhosis +celecoxib group and the control group (*P* > 0.05).

### Celecoxib inhibited SSTR-2 expression in L02 hepatocytes

**Figure 3:**
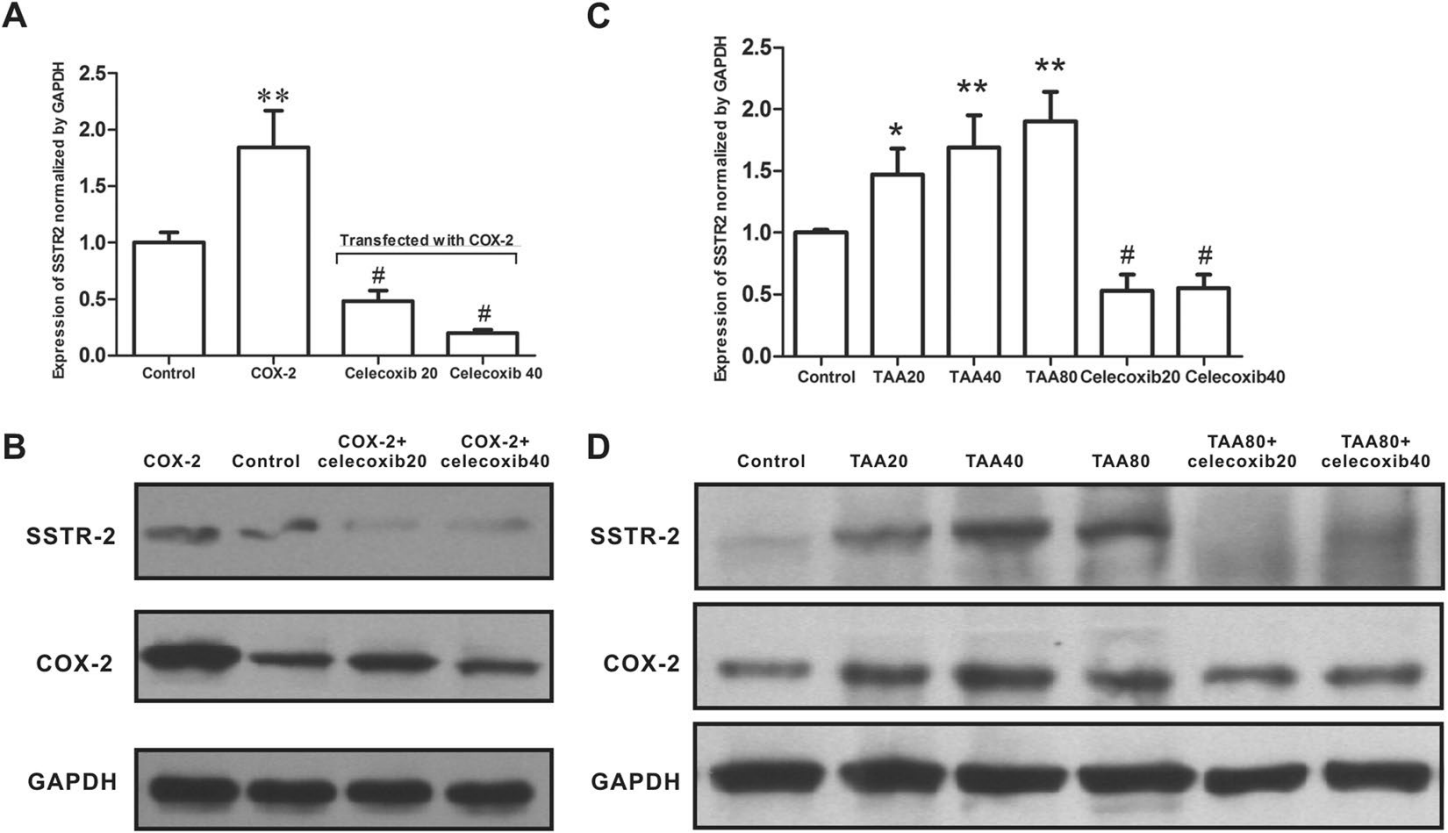
Enhanced SSTR-2 expression by COX-2 overexpression and the TAA treatment. (**A)** Relative level of SSTR-2 expression in L02 cells transfected with the COX-2 overexpression plasmid following the addition of celecoxib at a final concentration of 20 or 40 μM (Western blot). Control group: L02 cells transfected with the empty pcDNA3.1 plasmid; COX-2 group: L02 cells transfected with the COX-2 overexpression plasmid; COX-2+ celecoxib20 group: L02 cells transfected with the COX-2 overexpression plasmid and treated with 20 μM celecoxib; COX-2+ celecoxib40 group: L02 cells transfected with the COX-2 overexpression plasmid and treated with 40 μM celecoxib. ^**^P < 0.01 compared with the control group, ^#^*P* < 0.01 compared with the COX-2 group. (**B**) Western blots showing SSTR-2 and COX-2 levels in each group. The group of blots was cropped from different gels. (**C)** Western blot analysis of cells treated with final concentrations of 20, 40, or 80 mg/L TAA or the combination of 80 mg/L TAA and celecoxib at final concentrations of 20 or 40 μM. Control: L02 cells treated with vehicle; TAA20, TAA40 and TAA80: L02 cells treated with 20, 40, and 80 mg/L TAA, respectively; TAA80+ celecoxib20: L02 cells treated with TAA (80 mg/L) plus celecoxib (20 μM); TAA80+ celecoxib40: L02 cells treated with TAA (80 mg/L) plus celecoxib (40 μM). ^*^P < 0.05 compared with the control group. ^**^P < 0.01 compared with the control group; ^#^*P* < 0.01 compared with the TAA80 group. (**D**) Western blots showing SSTR-2 and COX-2 levels in each group. n = 3 samples per group. The group of blots was cropped from different gels.

To investigate the role of COX-2 in the regulation of SSTR-2 expression, we induced COX-2 expression in L02 hepatocytes by transfecting cells with a COX-2 overexpression plasmid or by incubating cells with TAA. The COX-2 gene was successfully cloned into the pcDNA3.1 expression plasmid, which was confirmed by DNA sequencing. COX-2-pcDNA3.1 was then transfected into L02 cells and was confirmed by Western blotting for COX-2. A significantly higher level of the SSTR-2 protein was observed in L02 cells transfected with COX-2-pcDNA than in cells transfected with empty-pcDNA3.1 (*P* < 0.01). However, this up-regulation was dramatically suppressed by the addition of celecoxib at concentrations of 20 μM and 40 μM (*P* < 0.01; Fig. 3A,B). Consistent with these findings, levels of the SSTR-2 and COX-2 proteins were both significantly increased in L02 cells incubated with TAA in a dose-dependent manner (*P* < 0.05). Similarly, this TAA-induced (80 mg/L) up-regulation of SSTR-2 was also inhibited by the addition of celecoxib (*P* < 0.01; Fig. 3C,D).

### COX-2 up-regulated SSTR-2 via the PKC-CREB signaling pathway

**Figure 4:**
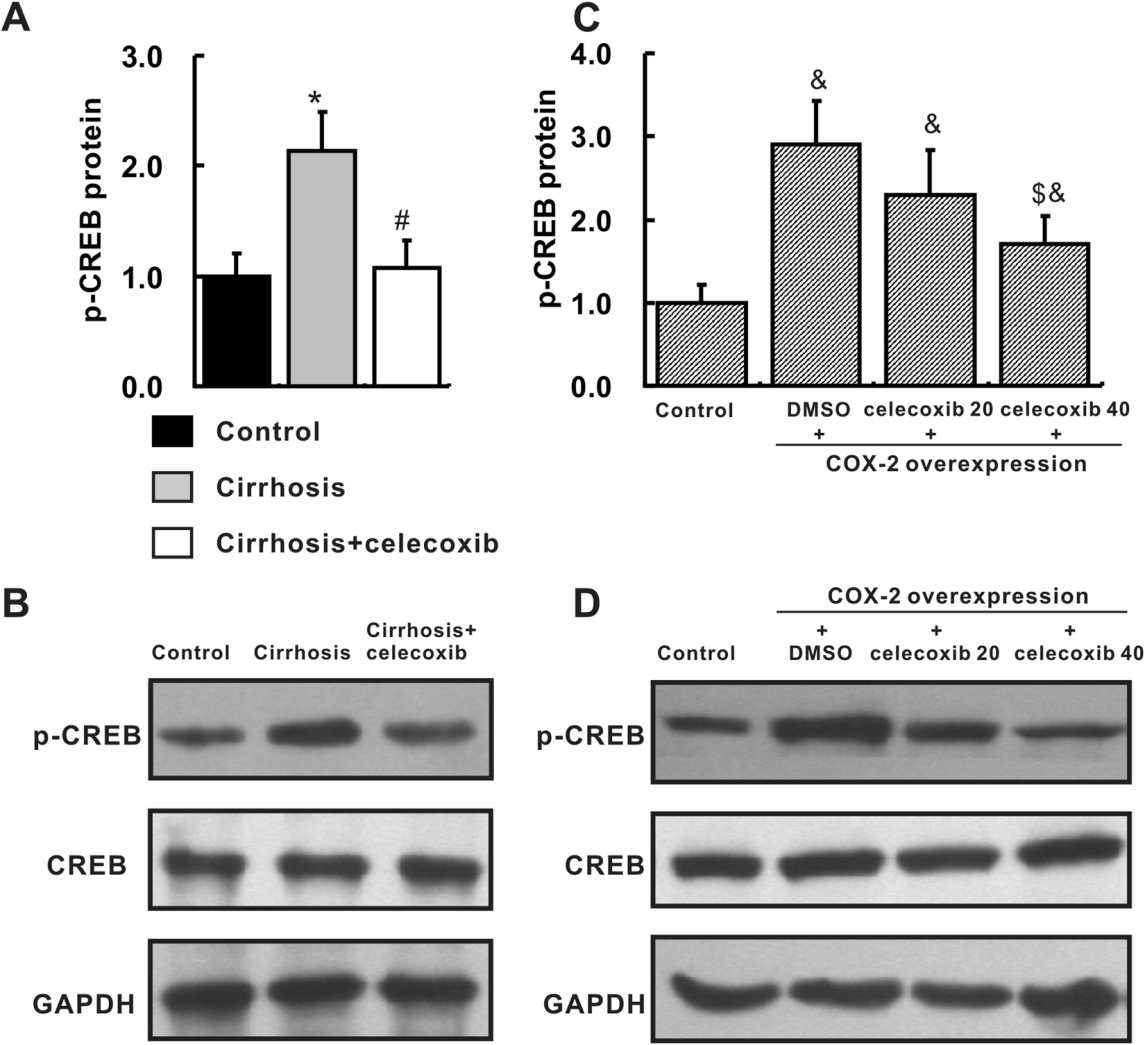
Increased p-CREB levels in cirrhotic livers and COX-2-transfected L02 cells. (**A**,**B**) Levels of p-CREB in the liver, as determined by Western blotting, n = 6 samples per group. The group of blots was cropped from different gels. ^*^P < 0.05 compared with the control group, ^#^*P* < 0.05 compared with the cirrhosis group. (**C**,**D**) Levels of p-CREB in COX-2-transfected L02 cells, as quantified by Western blotting. Control group: L02 cells transfected with the empty pcDNA3.1 plasmid; other three groups: L02 cells transfected with the COX-2 overexpression plasmid and treated with DMSO, or celecoxib (20 μM or 40 μM), respectively. The group of blots was cropped from different gels. ^&^*P* < 0.05 compared with the control cells. ^$^*P* < 0.05 compared with DMSO-treated, COX-2-transfected cells. n = 3 samples per group.

**Figure 5:**
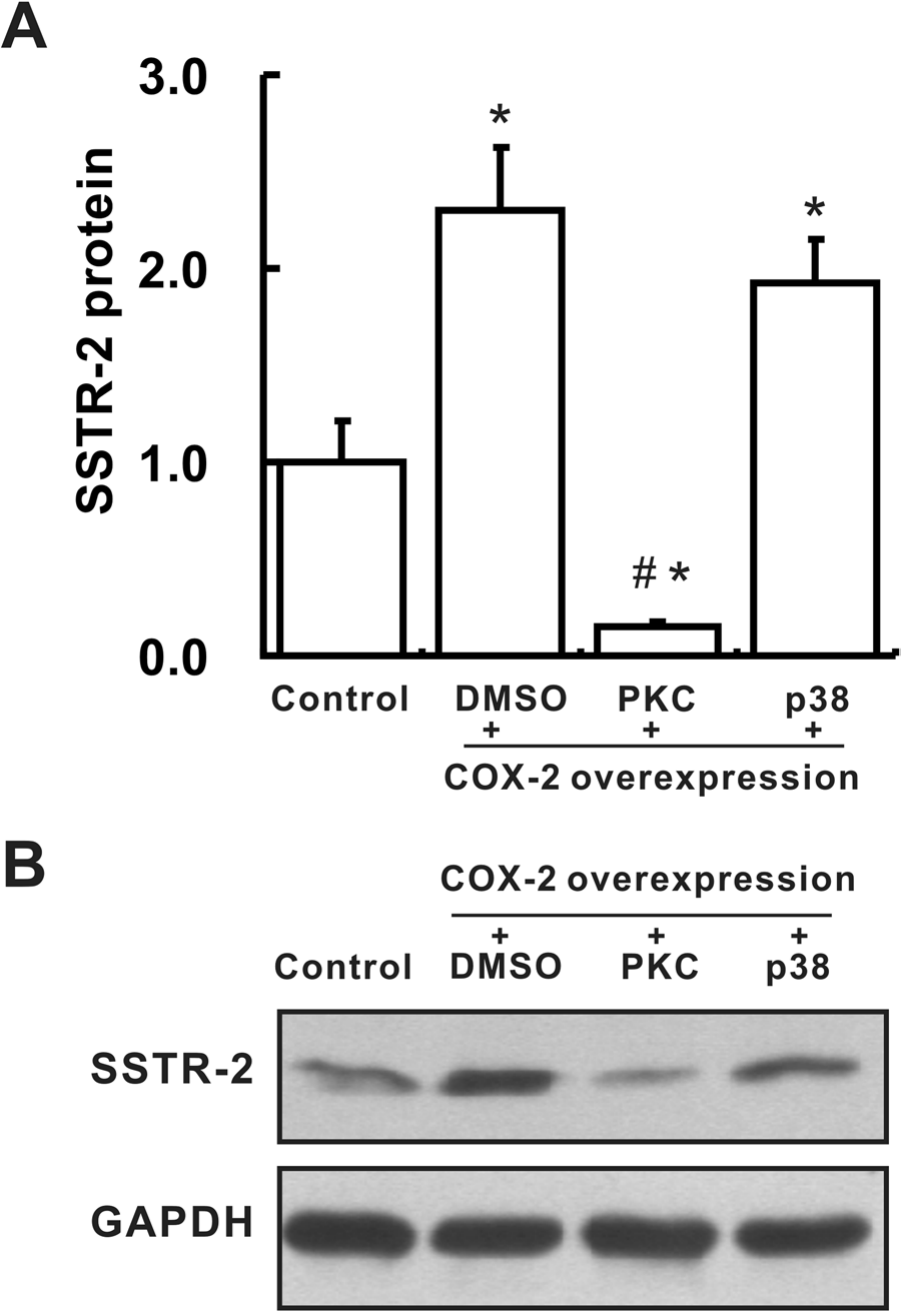
COX-2 up-regulates SSTR-2 expression via the PKC/p38–CREB signaling pathway. (**A**,**B**) Levels of SSTR-2 in L02 cells, as determined by Western blotting. Cells were treated with DMSO or p38 (2 μM) or PKC (2 μM) inhibitors 4 hours after transfection with the COX-2 overexpression plasmid. Cells in the control group were transfected with the empty pcDNA3.1 plasmid. n = 3 samples per group. The group of blots was cropped from different gels. ^*^P < 0.05 compared with the control cells, ^#^*P* < 0.05 compared with DMSO-treated L02 cells that were transfected with the COX-2 overexpression plasmid.

The cAMP response element binding protein (CREB) is an important transcription factors that binds to the promoter region of the SSTR-2 gene. To investigate the possible mechanisms by which SSTR-2 expression was up-regulated in the cirrhotic liver, we detected CREB levels in cirrhotic livers and L02 cells. *In vivo*, levels of phosphorylated CREB (p-CREB) were markedly increased in the cirrhosis group compared with those in the control group. However, the increased p-CREB level was significantly reduced in the celecoxib group (Fig. 4A,B). *In vitro*, p-CREB levels were significantly increased in L02 cells transfected with the COX-2 overexpression plasmid compared with the levels in the control group, and this change was inhibited by the addition of celecoxib in a dose-dependent manner (Fig. 4C,D). P-CREB is an important downstream transcription factor of the PKC and p38 signaling pathways. The PKC and p38 pathways were then inhibited by selective inhibitors in L02 cells transfected with COX-2. The PKC inhibitor, but not the p38 inhibitor, significantly decreased the up-regulation of SSTR-2 expression induced by COX-2 overexpression (Fig. 5). Based on these results, COX-2 might up-regulate SSTR-2 expression via the PKC-CREB pathway.

### DNA methylation of the SSTR-2 gene remained unchanged in rats with liver cirrhosis

**Figure 6:**
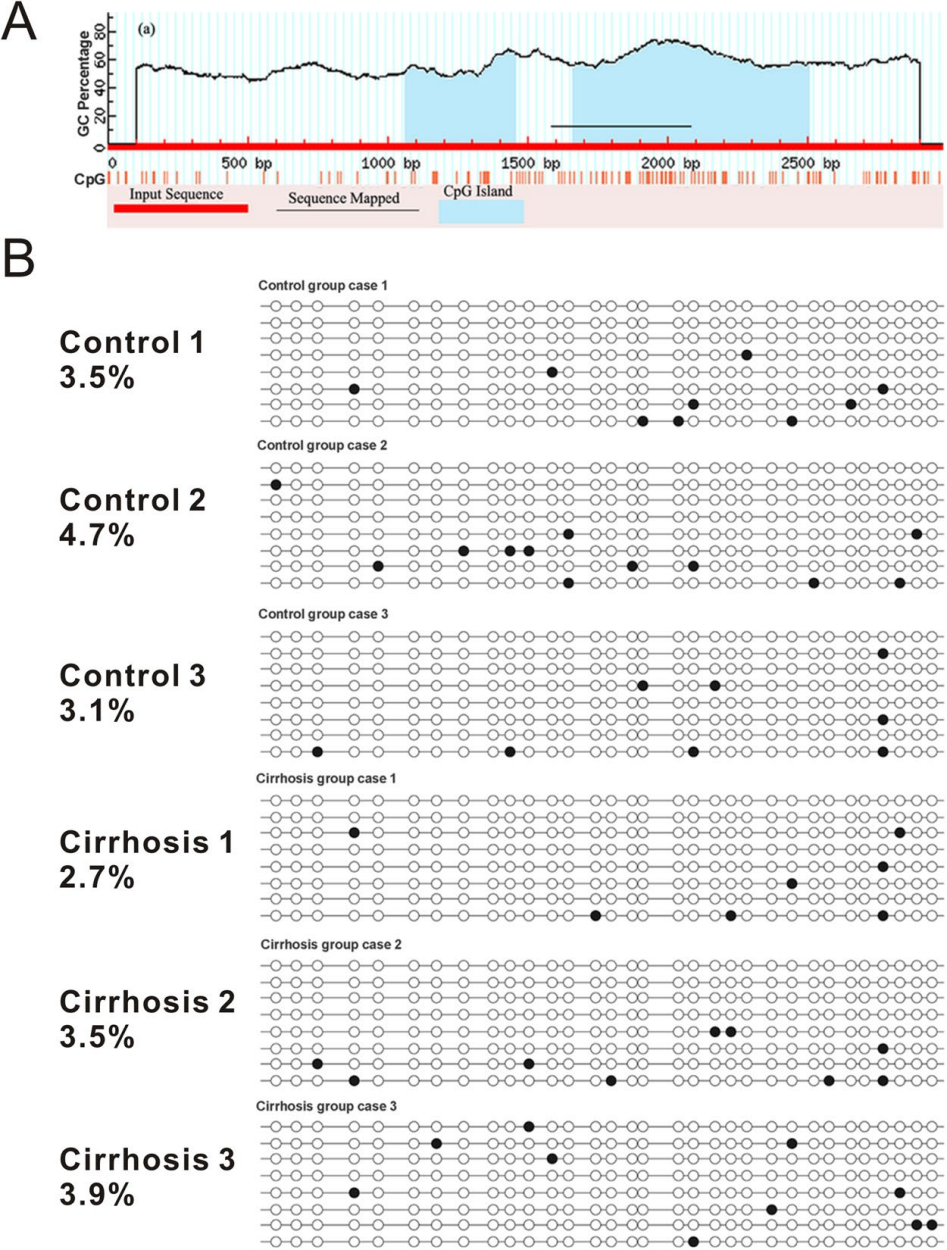
DNA methylation status of the SSTR-2 gene, as determined by the Sequenom MassARRAY. The horizontal axis represents −2000 to +1000 bp relative to the transcriptional start site of the SSTR-2 gene, while the vertical axis represents the DNA methylation level. No significant difference in methylation levels was observed between the control group and the cirrhosis group.

**Figure 7:**
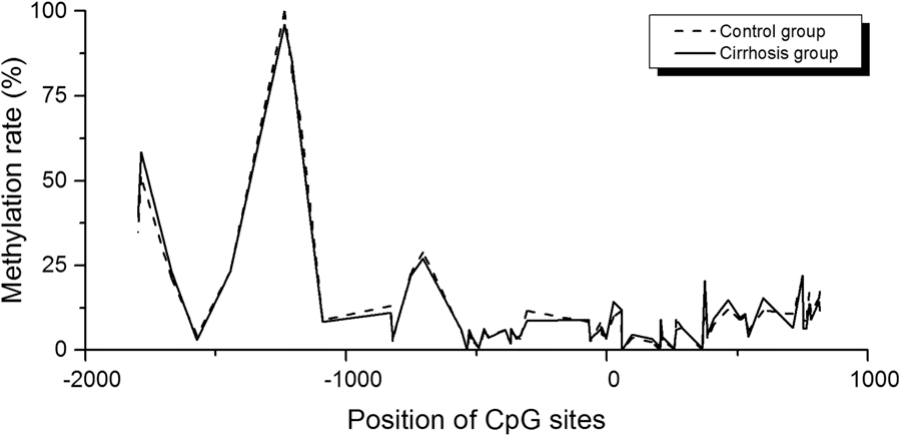
Analysis of the DNA methylation status of the SSTR-2 gene, as determined by bisulfite sequencing. (**A**) Results of the analysis of CpG islands in the SSTR-2 gene (−2000 to +1000). (**B**) DNA methylation level of the SSTR-2 gene (−413 to +85) in liver tissues from the control group and cirrhosis group. Solid and open circles represent methylated and unmethylated CpG dinucleotides, respectively. The numbers on the left show the methylation level of each sample.

Sequences between −2000 and +1000 relative to the transcription start site were analyzed using MethPrimer. Two CpG islands located between −939 to −545 and −337 to +590, respectively, were identified. Methylation levels of 49 CpG sites between −2000 and +1000 were analyzed using the Sequenom MassARRAY EpiTYPER. The methylation levels of the distal region (−2000 to −1200) were relatively high, while the methylation levels of the proximal region (−1200 to +1000) tended to be low. Overall, no significant difference in methylation levels was observed between the control group and the cirrhosis group (Fig. 6). A DNA fragment of 499 bp containing 32 CpGs located within the regulatory area (−413 to +85) was further analyzed using bisulfite sequencing. Consistently, low methylation levels were detected in the control group (3.7%) and the cirrhosis group (3.3%; *P* > 0.05, Fig. 7).

## Discussion

Previous studies have demonstrated that the expression of SSTR-2 can be differentially regulated by hormones (estrogen, testosterone and glucocorticoids), inflammatory cytokines (IL-1α and IL-1β), epigenetics (DNA methylation and histone acetylation), lipopolysaccharides and peptidoglycans^15,21,27^. In the present study, hepatic SSTR-2 expression was up-regulated in the TAA-induced cirrhotic rat model. Furthermore, COX-2 might play a role in this process via the PKC-CREB signaling pathway *in vivo* and *in vitro*. Since comparable DNA methylation levels were observed upstream of the hepatic SSTR-2 promoter region in the normal and cirrhotic livers, DNA methylation may not contribute to the up-regulation of SSTR-2 expression in the cirrhotic rat liver.

A cirrhotic rat model was established by an intraperitoneal injection of TAA, a hepatotoxic chemical, to investigate the mechanism regulating SSTR-2 expression in the cirrhotic liver. Consistent with a previous study, hepatic SSTR-2 expression was up-regulated in the liver cirrhosis group^10^. The DNA methylation level in the promoter region of the SSTR-2 gene was reported to be inversely correlated with its expression level in several cancer cell lines, and DNA methylation silences the SSTR-2 promoter *in vitro*^15^. Based on accumulating evidence, aberrant global DNA methylation contributes to the progression of liver cirrhosis^28–30^. Specific loci, including collagen 1A1, secreted phosphoprotein 1, SPRR3 and TNFSF15, have been confirmed to be hypomethylated, resulting in the up-regulation of these genes in patients with liver cirrhosis of various etiologies^29–31^. However, researchers have not determined whether DNA methylation causes SSTR-2 up-regulation in the liver of a cirrhotic rat.

Accordingly, the Sequenom MassARRAY assay and bisulfite sequencing were then used to detect the methylation level upstream of the SSTR-2 gene. However, no significant difference in methylation level of the SSTR-2 gene was observed within −2000 to +1000 relative to the transcriptional start site using the Sequenom MassARRAY assay. Since the Sequenom MassARRAY assay was not able to examine every CpG site in the detected region, the bisulfite sequencing assay was further applied to detect the region from −413 to +85, which contains most of the regulatory elements in the SSTR-2 promoter^32^. Nevertheless, similar to the results from the Sequenom MassARRAY, no significant difference was observed between the cirrhosis group and the control group. Although the reason is not quite clear, the difference in the cell proliferation state between the cirrhotic liver and cultured human hepatocytes might explain this discrepancy. Notably, the sample size for the methylation analysis (3 per group) in the present study was sufficient to detect a 1% difference between the two groups. Torrisani J identified two CpG islands and a new transcription start site located 3.8 kb upstream (−3939 to −4205)^15^. Hence, NCBI has updated the transcription start site and defined Torrisani J.’s upstream transcription start site as +1. By performing a CpG analysis using the Methyl primer express software, we found that most CpG islands were located within −2000 to +1000 in rats (Fig. 6A). Importantly, Torrisani J also verified that the new transcription start site is located in close proximity to a region in which the transcription start site is located in rat^15^. Thus, the promoter region analyzed in the present study covered the upstream promoter region reported by Torrisani J. Accordingly, the DNA methylation at the promoter region may not regulate SSTR-2 expression in the liver of a cirrhotic rat. However, further study is needed to determine whether DNA methylation at other sites regulates SSTR-2 expression.

We previously explored the therapeutic efficacy of celecoxib for liver cirrhosis and found that celecoxib attenuated TAA-induced liver cirrhosis by inhibiting fibrosis, angiogenesis, inflammation and the epithelial-to-mesenchymal transition of hepatocytes in animal models^23–25^. In the present study, SSTR-2 expression was inhibited by the addition of celecoxib, a COX-2 inhibitor that functions by directly binding to the active site of COX-2. In one previous study, which investigated the correlation between the expression of SSTR2 and COX-2, the author applied an immunochemistry method to examine the expression of COX-2 and SSTR2 in gastroenteropancreatic neuroendocrine tumors. Interestingly, an inverse correlation was identified between the expression of COX-2 and SSTR2 in the foregut, but not the hindgut, in subjects with neuroendocrine tumors^33^. However, the limitations of this study were the exclusive use of the immunochemistry method and the correlation coefficient was set at a low level of 0.3. In the present study, COX-2 induced SSTR-2 expression *in vitro*. We noticed an up-regulation of SSTR-2 expression when COX-2 expression was induced, and this up-regulation was diminished when the COX-2 signaling pathway was inhibited by celecoxib. In our study and a previous study, SSTR-2 was mainly expressed in hepatocytes of cirrhotic livers. COX-2 was also mainly expressed in hepatocytes and macrophages^10,34^. Accordingly, strategies targeting the COX-2/SSTR-2 signaling pathway are postulated to either promote liver fibrosis by reducing hepatic inflammation or improve liver fibrosis by inhibiting the epithelial-to-mesenchymal transition of hepatocytes.

Activating transcription factor-2 (ATF-2), CREB and c-Jun are important transcriptional factors that directly bind to the ATF/CRE site within the SSTR-2 promoter region, thereby activating SSTR-2 transcription^32,35^. Levels of p-CREB and c-Jun are both increased in cirrhotic livers or in activated HSCs^36,37^. Meanwhile, COX-2 increases the phosphorylation of CREB and c-Jun by activating several signaling pathways^38^. Consistent with previous studies, we also observed increased p-CREB levels in cirrhotic livers, a change that was inhibited by the addition of celecoxib. Furthermore, the COX-2-PKC-CREB signaling pathway might participate in the mechanism regulating SSTR2 expression *in vitro*. Based on these data, COX-2 might up-regulate hepatic SSTR2 expression in the cirrhotic liver via the PKC-CREB signaling pathway. Notably, our study only concentrated on the role of one signaling pathway in the mechanism regulating SSTR-2 expression, and further studies are required to determine whether other pathways are involved in this process.

Based on results of the present study, COX-2 inhibitors may influence the therapeutic efficacy of SST or its analogues, particularly when their effects are mediated by SSTR-2. A combination of a COX-2 inhibitor and an SST or its analogues is frequently employed as a treatment for pancreatitis and various tumors^39,40^. In a mouse model of pancreatitis, the administration of octreotide in combination with diclofenac sodium (a COX inhibitor) did not show superior benefits compared to the administration of each agent separately^41^. The author suggested that this lack of change may be due to the interaction between the two agents. Diclofenac sodium may have inhibited the anti-secretory effect of octreotide on the exocrine pancreas in this model. In contrast to this finding, one of our previous studies found that nude mice with HepG2 xenografts that were treated with SOM230 and celecoxib together exhibited better survival than mice given SOM230 or celecoxib alone^42^. As shown in our previous study, celecoxib and the SST analogue octreotide synergistically ameliorate portal hypertension and liver fibrosis in the cirrhotic liver by inhibiting angiogenesis^3^. The probable explanation may be that the two agents exert their anti-tumor and anti-fibrosis effects through similar mechanisms, including inhibiting proliferation and angiogenesis and promoting apoptosis^3,27,43^. Moreover, the inhibition of SSTR-2 expression by celecoxib might lead to the synergistic therapeutic effects of celecoxib and octreotide on HepG2 xenografts and cirrhotic rats.

**Figure 8:**
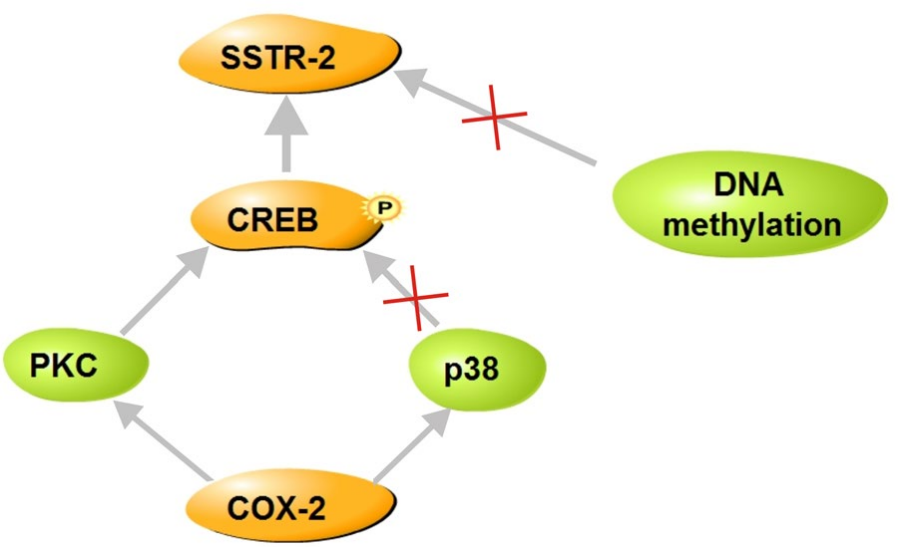
Schematic of mechanism by which COX-2 regulates SSTR-2 expression. COX-2 up-regulate SSTR-2 expression in the cirrhotic liver. The presumable mechanism is that CREB may participate in this regulatory process via the PKC signaling pathway, while DNA methylation and the p38 signaling pathway are not likely involved in this process.

In conclusion, as depicted in Fig. 8, COX-2 up-regulates hepatic SSTR-2 expression in a rat liver cirrhosis model via the PKC-CREB signaling pathway, while DNA methylation is not likely responsible for this process.

## Methods

### Animal model

Eighteen male Sprague-Dawley (SD) rats (Chengdu Dossy Experimental Animals Co., Chengdu, China) weighing between 180 and 200 g were housed at 25 °C on a 12 hour light-dark cycle. Rats were randomized into three groups, with six rats in each group. Rats in the cirrhosis group were intraperitoneally injected with 200 mg/kg TAA (Sigma Chemical Co., St. Louis, MO, USA) every three days for 16 weeks; the control group received an equivalent volume of saline. Rats in the cirrhosis +celecoxib group were intraperitoneally injected with TAA combined with the intragastric administration of 20 mg/kg/day celecoxib (Pfizer, New York, NY, USA) after the TAA injection. After an additional 16 weeks, animals were sacrificed under anesthesia. Livers were removed for further experiments. All procedures involved in the animal studies were approved by the Animal Use and Care Committee of Sichuan University and were conducted according to the regulations established by Sichuan University.

### Histological studies

Livers were fixed with 4% neutral buffered paraformaldehyde, embedded in paraffin, sectioned consecutively at a thickness of 3 μm and stained with Masson’s trichrome (MT). The degree of fibrosis was assessed using Ishak’s scoring system.

### IHC staining

Sections were deparaffinized in xylene and serial ethanol dilutions. Antigen retrieval was performed by heating the sections in 10 mM sodium citrate buffer. Sections were blocked with 10% goat serum and incubated with the primary antibody overnight at 4 °C. SSTR-2 (1:100, Santa Cruz Biotechnology, Santa Cruz, CA, USA) and COX-2 (1:200, Abcam, Cambridge, UK) antibodies were used. After an incubation with biotinylated secondary antibodies and the streptavidin-biotin-complex, sections were stained with a solution of 3,3-diaminobenzidine tetrahydrochloride and counterstained with hematoxylin.

### Protein extraction and Western blotting

Proteins were extracted from liver tissues or cultured cells using a protein extraction kit (Keygen Biotec, Nanjing, China), according to the manufacturer’s protocol. Protein concentrations were determined using a BCA protein assay kit (Biotime, Shanghai, China). Next, each sample was mixed with 6× SDS sample buffer and boiled for 3 min. Total proteins (20 μg) were separated on 8% SDS-PAGE gels and electrotransferred to polyvinylidene fluoride membranes (Millipore, Billerica, MA, USA). Membranes were incubated with appropriate primary antibodies overnight at 4 °C and then incubated with horseradish peroxidase-conjugated secondary antibody (ZSGB-bio, Beijing, China) at room temperature for two hours. Enhanced chemiluminescence (Biotime) was used to visualize the protein bands. Protein expression was normalized to GAPDH. The SSTR-2 antibody was obtained from Santa Cruz Biotechnology, Santa Cruz, CA, USA; the COX-2 antibody was obtained from Biworld Technology, Co., Nanjing, China; CREB and p-CREB antibodies were obtained from ImmunoWay Biotechnology Co., Newark, DE, USA; and the GAPDH antibody was obtained from Goodhere, Hangzhou, China.

### RNA extraction and quantitative real-time PCR (qRT-PCR)

Total RNA was extracted from tissues and cultured cells using TRIzol (Invitrogen, Carlsbad, CA, USA) and reverse transcribed to cDNAs (Fermentas, Ontario, Canada) according to the manufacturer’s protocols. Quantitative real-time PCR was performed using a QuantiFast SYBR Green PCR kit (Qiagen, Valencia, CA, USA) in a CFX96 real-time PCR detection system (Bio-Rad, Hercules, USA). The amplification conditions were consistent with manufacturer’s recommendations, with an initial heat activation at 95 °C for 5 min followed by 40 cycles of denaturation at 95 °C for 10 s, and annealing at 60 °C for 30 s. All primers were synthesized by Invitrogen Co. (Shanghai, China). Primer sequences are shown in Table 1. Gene expression was normalized to GAPDH.

**Table 1:** Sequences of primers used in this study.

Target	Sequence	Size (bp)
Rat SSTR-2	F: 5′-GAAAAGCAAGATGTCACGATAG-3′ R: 5′-TTGGCTCCCATTGAACTG-3′	135
Rat COX-2	F: 5′-GCTCATACTGATAGGAGAGACGA-3′ R: 5′-TGGAACTGCTGGTTGAAAAG-3′	117
Rat GAPDH	F: 5′-TCGGTGTGAACGGATTTG-3′ R: 5′-CTCAGCCTTGACTGTGCC-3′	173
Bisulfite sequencing	F: 5′-AAACTACCCTAACCTATAAATCAT-3′ R: 5′-GGTTTTGTAATTTGTGTTTTGTT-3′	499
Sequenom MassARRAY (1)	F: 5′-GGGGTTTTTATTTATTTATTGGGGT-3′ R: 5′-TAAACTCAAACCTCCTCCTACCTCT-3′	487
Sequenom MassARRAY (2)	F: 5′-TTGAGGAGATTTGAATTTAGAATGG-3′ R: 5′-AAAAAACCAAAAAATAAAAACAACC-3′	356
Sequenom MassARRAY (3)	F: 5′-TTGTTAGTTATTGTTTGTTGGTTTGA-3′ R: 5′-CCACCCAAATACACTCTCTATCTCT-3′	347
Sequenom MassARRAY (4)	F: 5′-GTTGGGGTTGGGTTAGATTAGTAAG-3′ R: 5′-TTTTACCCAACAAATTACAAACAACT-3′	358
Sequenom MassARRAY (5)	F: 5′-GGTGTTTAAAGTGTGTTGTTTTTTTT-3′ R: 5′-CTCCTCACCAAATCCTACAAAACT-3′	470
Sequenom MassARRAY (6)	F: 5′-GTAGGGAGGGATAATATATTTTGGG-3′ R: 5′-ACACTTAACATACCCCTTCTCCTTT-3′	339

### Construction of the COX-2 expression vector

The human COX-2 gene was synthesized and cloned into the pcDNA3.1 expression vector (Genewhiz, Jiangsu, China) which was then transformed into *E. coli* and extracted using a plasmid mini kit (Omega).

### Cell culture, treatment and transfection

Human h L02 cells (the Type Culture Collection of the Chinese Academy of Sciences, Shanghai, China) were cultured in RPMI-1640 media containing 10% fetal bovine serum (ScienCell, San Diego, CA) and maintained in a humidified atmosphere containing 5% CO_2_ at 37 °C. Transfections were performed using jetPEI (PolyPlus Transfection, Illkirch, France), according to the manufacturer’s protocol. Four hours after transfection, cells transfected with the COX-2 plasmid were resuspended in medium containing celecoxib at a final concentration of 20 or 40 μM. For the TAA treatment, cells were incubated in medium containing TAA at a final concentration of 20 mg/L, 40 mg/L, or 80 mg/L or a combination of TAA (final concentration of 80 mg/L) and celecoxib (final concentration of 20 μM or 40 μM). For the experiments using signaling pathway inhibitors, cells were treated with p38 (2 μM) and PKC (2 μM) inhibitors at the indicated concentrations. The inhibitors were obtained from Selleck Chemicals (Shanghai, China). Twenty-four hours after treatment, mRNA and proteins were harvested from cells for further study.

### Detection of DNA methylation levels using the Sequenom MassARRAY

A 3 kb DNA sequence from −2000 to +1000 of the SSTR-2 (GeneBank NC_005109.4, 102136283–102143449) transcriptional start site was subjected to an analysis of CpG islands using MethPrimer (http://www.urogene.org/methprimer) with the following parameters: island size > 200 bp, GC Percent > 50.0, Obs/Exp > 0.6. Genomic DNA was extracted using the Blood and Tissue DNA purification kit (Qiagen) and subjected to bisulfite modification using EpiTect Bisulfite Kits (Qiagen) according to the manufacturer’s protocol. Primers were designed using MethPrimer (http://www.urogene.org/methprimer/) and Primer Premier 5.0 (Premier, Canada), as shown in Table 1. Sequences of interest were then amplified using HotStar Taq DNA Polymerase (Qiagen). The Sequenom MassARRAY was followed by *in vitro* RNA transcription and base-specific cleavage (MassCCLEAVE Kit, San Diego, USA, SEQUENOM), according to the manufacturer’s protocol. Mass spectra were analyzed using the MassARRAY Compact System (SEQUENOM). Methylation ratios were then generated by the EpiTYPER software (SEQUENOM). The Sequenom methylation analysis was performed at CapitalBio, Beijing, China.

### Detection of DNA methylation levels by bisulfite sequencing

For bisulfite sequencing, the PCR product was cloned into PMD 19-T vector (Takara, Dalian, China), transformed into *E. coli* and grown on LB agar plates, containing 100 μg/mL ampicillin. Positive colonies were selected, and the plasmid was extracted using a plasmid mini kit (Omega Bio-tek, Norcross, USA). Eight clones were randomly selected and both strands were sequenced (Invitrogen).

### Statistical analysis

Quantitative data are presented as means ± standard deviations and analyzed using SPSS 13.0. Analysis of variance (ANOVA) and the Student-Newman-Keuls (SNK) analysis were used for multiple comparisons. Student’s *t* test was used for comparisons between two groups. Cell-based experiments were performed in triplicate. The difference was considered statistically significant when *P* < 0.05.
